# FLNC Associada a Cardiomiopatia Restritiva e Hipertrabeculação, uma Associação Rara

**DOI:** 10.36660/abc.20230790

**Published:** 2024-06-06

**Authors:** Ana M. Aristizabal, Carlos Alberto Guzmán-Serrano, María Isabel Lizcano, Walter Mosquera, Juliana Lores, Harry Pachajoa, Cesar Cely

**Affiliations:** 1 Universidad Icesi Facultad de Ciencias de la Salud Cali Colômbia Facultad de Ciencias de la Salud, Universidad Icesi, Cali – Colômbia; 2 Fundación Valle del Lili Departamento de Cardiología Pediátrica Cali Colômbia Departamento de Cardiología Pediátrica - Fundación Valle del Lili, Cali – Colômbia; 3 Fundación Valle del Lili Centro de Investigaciones Clínicas Cali Colômbia Centro de Investigaciones Clínicas - Fundación Valle del Lili, Cali – Colômbia; 4 Hospital Universitario del Valle Departamento de Cardiología Pediátrica Cali Colômbia Departamento de Cardiología Pediátrica - Hospital Universitario del Valle, Cali – Colômbia; 5 Universidad Icesi Centro de Investigaciones en Anomalías Congénitas y Enfermedades Raras Cali Colômbia Centro de Investigaciones en Anomalías Congénitas y Enfermedades Raras (CIACER) - Universidad Icesi, Cali – Colômbia; 6 Pontificia Universidad Javeriana Cali Facultad de Ciencias de la Salud Departamento de Ciencias Básicas Cali Colômbia Departamento de Ciencias Básicas - Facultad de Ciencias de la Salud - Pontificia Universidad Javeriana Cali, Cali – Colômbia; 7 Fundación Valle del Lili Cali Colômbia Servicio de Genética - Fundación Valle del Lili, Cali – Colômbia

**Keywords:** Cardiomiopatia Restritiva, Genética, Filaminas

## Abstract

Menina de seis anos com cardiomiopatia restritiva e hipertrabeculação na qual, devido ao início precoce da doença, foi realizado sequenciamento completo do exoma, revelando a presença de uma nova variante heterozigótica missense no gene FLNC. A mesma variante genética também foi identificada em seu pai, que, já adulto, apresentava resultados de imagem normais e não apresentava sintomas. Esta variante não foi relatada em bancos de dados populacionais ou na literatura médica atual e é classificada como provavelmente patogênica.

## Introdução

A apresentação clínica da cardiomiopatia pode variar desde pacientes assintomáticos, com manifestações inespecíficas, até um curso progressivo e grave que pode resultar em choque cardiogênico, arritmias e até morte súbita.^1^ Embora a linhagem genética tenha sido identificada para algumas das cardiomiopatias, as formas familiares apresentam diversos modos de herança de acordo com o fenótipo expresso.^1^ A maior parte dos dados disponíveis sobre cardiomiopatias refere-se à população adulta. Neste relato, descrevemos o caso de um paciente em idade escolar, com associação de cardiomiopatia restritiva e hipertrabeculação, anteriormente conhecida como não-compactação de ventrículo esquerdo não dilatado (VEND).

## Relato de Caso

Uma menina de seis anos, previamente hígida, sem história familiar ou de consanguinidade relevante, apresentava história de hiporexia há 24 meses, aumento da circunferência abdominal e êmese recorrente; nos últimos dois meses com dor torácica e diminuição progressiva da classe funcional. Ela foi encontrada com hepatomegalia aproximadamente 6 cm abaixo da margem costal. O ecocardiograma inicial revelou dilatação acentuada das veias supra-hepáticas e veia cava inferior, com dilatação biatrial acentuada, disfunção diastólica com padrão restritivo e sinais indiretos de hipertensão pulmonar. Suspeitou-se de CMR e iniciou-se tratamento com diuréticos, carvedilol e enalapril, com melhora da classe funcional.

**Figura 1:**
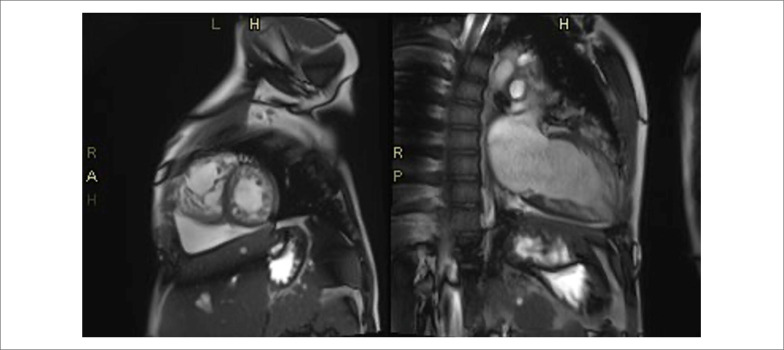
Ressonância magnética cardíaca: sequência cine SSFP TrusFisp em eixo longo 2 câmaras e eixo curto onde se observa hipertrabeculação significativa da cavidade ventricular esquerda, comprometendo as paredes lateral e inferior e médio-apical com relação região não compactada/região compactada de 4.

Foram solicitados estudos para esclarecer a etiologia da CMR, a ressonância magnética cardíaca relatou hipertrabeculação ventricular esquerda com relação região não compactada/região compactada de 4:1, disfunção sistólica, hipertrabeculação significativa da cavidade ventricular, dilatação do ventrículo direito com deterioração da função sistólica (Figura 1). Além disso, um ECG Holter de 24 horas descreveu alterações atriais direita e esquerda e distúrbio de repolarização nas derivações precordiais. Pelo exposto, a paciente foi discutida em reunião de cardiologia, considerando a coexistência de cardiomiopatia restritiva e hipertrabeculação.

Foi iniciado tratamento médico anti-insuficiência e houve melhora da classificação funcional da New York Heart Association (NYHA) de III para II. Porém, após alguns meses, a paciente apresentou piora clínica, necessitando de internação em unidade de terapia intensiva por insuficiência cardíaca refratária, sendo feito diagnóstico de tamponamento cardíaco necessitando de janela pericárdica, suporte com oxigenação mecânica extracorpórea e posterior hemorragia cerebral maciça levando à sua morte.

O consentimento informado por escrito foi obtido do responsável legal/parente mais próximo da menor, para publicar quaisquer imagens ou dados potencialmente identificáveis neste artigo. O comitê de ética aprovou a condução do estudo.

### Avaliação baseada em genômica

Devido ao diagnóstico de CMR de início precoce, a equipe médica de genética foi consultada. O exame físico era normal e não havia história familiar de cardiomiopatias ou morte súbita cardíaca. Após uma revisão cuidadosa do caso, foi realizado o sequenciamento completo do exoma (WES).

Uma nova variante heterozigótica missense no gene FLNC (NM_001458.5) foi identificada: c.7559C>A, p.Thr2520Asn e confirmada por sequenciamento Sanger. Essa substituição converte o códon treonina na posição 2520 em asparagina, localizado no domínio ROD2 no qual há agrupamento de variantes associadas principalmente à cardiomiopatia hipertrófica. Esta variante não foi relatada em bancos de dados populacionais ou na literatura médica atual e é classificada como provavelmente patogênica.

Outras variantes genéticas identificadas neste caso foram: uma variante frameshift heterozigótica no gene AGK (NM_018238.4): c.675delG, p.Trp225CysfsTer6, classificada como patogênica de acordo com as diretrizes da ACMG; e uma variante heterozigótica missense no gene PKP2 (NM_004572.4): c.1163G>A, p.Arg388Gln, classificada como variante de significado incerto (VSI).

**Figura 2:**
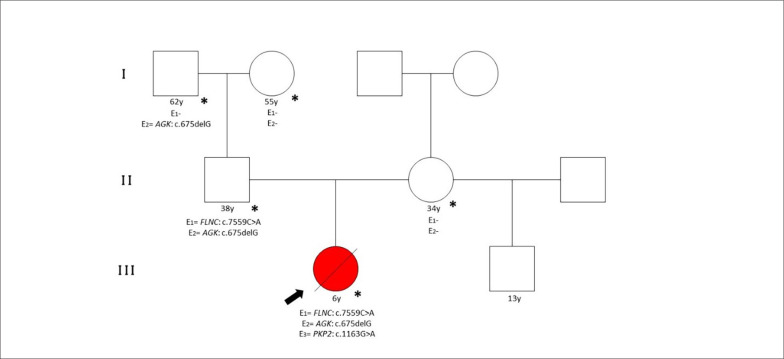
Informações de pedigree do paciente e familiares. Seta preta: o probando. Vermelha: Cardiomiopatia restritiva (*): Avaliação documentada. A nomenclatura padronizada de pedigree humano foi seguida (J Genet Counsel (2008) 17:424–433).

Nenhuma outra variante genética foi identificada neste caso. A mãe da paciente (34 anos), o pai (38 anos) e os avós paternos (55 e 62 anos) consentiram no teste genético, constatando que o pai era portador das variantes FLNC e AGK (Figura 2). Apresenta ecocardiograma normal e está em avaliação pela equipe de cardiologia.

## Discussão

No que diz respeito à CMR, os genes mais comumente implicados são aqueles que codificam proteínas sarcoméricas, incluindo aqueles para troponina I (TNNI3), cadeia pesada de β-miosina (MYH7), α-actina cardíaca (ACTC1), titina (TTN) e genes da cadeia leve da miosina.^2^ Existem casos esporádicos e familiares, e até 30% dos pacientes têm história familiar relevante, sendo a endomiocardiofibrose a causa mais frequente.^3^ Da mesma forma, a hipertrabeculação corresponde a um traço fenotípico e não a uma cardiomiopatia por si só.^1^ É caracterizada pela presença de trabéculas proeminentes no ventrículo esquerdo e recessos intertrabeculares profundos e também tem sido associada a genes sarcoméricos, mais comumente o gene MYH7.^1^ Portanto, discute-se se corresponde a uma cardiomiopatia diferente ou a um traço morfológico comum. Vale ressaltar que pacientes com variantes patogênicas nos genes do sarcômero tendem a apresentar início precoce e maior incidência de efeitos adversos.^4^ Outros genes ligados à hipertrabeculação são ZASP, distrobrevina e tafazzin, bem como um único ponto de mutação no gene da cadeia pesada da beta-miosina.^5^

Neste caso, a WES identificou 3 variantes diferentes: variantes dos genes FLNC, AGK e PKP2. Entre estas, a primeira variante foi de particular interesse. O gene FLNC codifica a proteína filamina C de ligação à actina, um dos três membros da família encontrados em humanos.^6^ A filamina C é composta por 6 domínios: um domínio de ligação à actina, subdomínios ROD1 e ROD2 e um domínio de dimerização no seu terminal C; e serve como uma âncora de proteínas do sarcolema para o citoesqueleto e sistema de ligação células-células ou células-matriz extracelular.^6,7^

Mutações no gene FLNC foram inicialmente associadas à estrutura e função do tecido muscular esquelético, embora mais recentemente tenham sido reconhecidas em CMD, CMH, VENC e CMR.^6^ Maior prevalência de mutações foi encontrada em pacientes com CMD, seguido de CMH.^7^ Poucos casos foram notificados ao CMR, com variantes localizadas principalmente nos domínios ROD1 e ROD2; a variante encontrada neste caso está localizada em ROD2.^7–9^ Outros relatórios mostraram variantes não repetitivas descritas no gene FLNC entre populações pediátricas e adultas, fornecendo informações que sugerem uma ligação familiar.^10,11^ No nosso caso, foi identificada uma nova mutação missense, enquanto um estudo de segregação familiar revelou o pai como portador da mesma variante, sendo atualmente um adulto assintomático de 38 anos. Mais estudos são necessários para atribuir significado patogênico a esta mutação e sua relação com a herança familiar.

Em relação às variantes de AGK e PKP2, a primeira geralmente é encontrada na síndrome de Sengers e em um tipo de catarata autossômica recessiva; como a paciente apresenta essa mutação em estado heterozigoto, ela é considerada portadora.^12^ Este último, PKP2, uma variante heterozigótica patogênica que tem sido associada à cardiomiopatia arritmogênica; neste caso, a variante é classificada como VSI e não se correlaciona com o fenótipo encontrado, mas pesquisas estão em andamento na área de genética cardiovascular para compreender as possíveis contribuições de mutações genéticas de baixa a média penetrância nas cardiomiopatias.^13–15^

Tal como evidenciado na nossa paciente, apesar do início do tratamento médico, ela apresentou rápida deterioração do seu quadro clínico devido a insuficiência cardíaca refratária com desfecho fatal. Seguindo as diretrizes da Sociedade Europeia de Cardiologia, estudos genéticos poderiam ser realizados para confirmar o diagnóstico, avaliar o prognóstico, selecionar o tratamento, como parte do aconselhamento reprodutivo, ou quando o estudo visa fornecer informações necessárias para um parente próximo, em nossa opinião, é deve ser realizado em todos os pacientes cuja resposta terapêutica não esteja produzindo resultados, na presença de deterioração de início rápido ou na presença de múltiplos tipos de cardiomiopatias coexistentes. Portanto, consideramos relevante divulgar esse tipo de associação, uma vez que foi descrita uma variante potencialmente patogênica da FLNC nunca descrita anteriormente, o que poderia explicar sua evolução e contribuir para novos estudos sobre o tema.
