# Supplementary material for: FLNC Associada a Cardiomiopatia Restritiva e Hipertrabeculação, uma Associação Rara
Source: Arq Bras Cardiol. 2024 Jun 6;121(5):e20230790. [Article in Portuguese] doi: 10.36660/abc.20230790 (PMC11216340; doi:10.36660/abc.20230790)
Supplement: Translation

# Translation: FLNC Associated Restrictive Cardiomyopathy and Hypertrabeculation, a Rare Association

**DOI:** 10.36660/abc.20230790i

**Authors:** Ana M. Aristizabal, Carlos Alberto Guzmán-Serrano, María Isabel Lizcano, Walter Mosquera, Juliana Lores, Harry Pachajoa, Cesar Cely

**Affiliations:** 1 Cali Colombia Facultad de Ciencias de la Salud, Universidad Icesi, Cali – Colombia; 2 Cali Colombia Departamento de Cardiología Pediátrica - Fundación Valle del Lili, Cali – Colombia; 3 Cali Colombia Centro de Investigaciones Clínicas - Fundación Valle del Lili, Cali – Colombia; 4 Cali Colombia Departamento de Cardiología Pediátrica - Hospital Universitario del Valle, Cali – Colombia; 5 Cali Colombia Centro de Investigaciones en Anomalías Congénitas y Enfermedades Raras (CIACER) - Universidad Icesi, Cali – Colombia; 6 Cali Colombia Departamento de Ciencias Básicas - Facultad de Ciencias de la Salud - Pontificia Universidad Javeriana Cali, Cali – Colombia; 7 Cali Colombia Servicio de Genética - Fundación Valle del Lili, Cali – Colombia

**Keywords:** Restrictive Cardiomyopathy, Genetics, Filamins

## Abstract

A six-year-old girl with restrictive cardiomyopathy and hypertrabeculation, due to the early onset of her disease, whole exome sequencing was conducted, revealing the presence of a novel heterozygous missense variant in the FLNC gene. The same gene variant was also identified in her father, who, at an adult age, displayed normal imaging results and was symptom-free. This variant has not been reported in population databases or current medical literature and is classified as likely pathogenic.

## Introduction

Cardiomyopathy clinical presentation can range from asymptomatic patients, with nonspecific manifestations, up to a progressive and severe course that can result in cardiogenic shock, arrhythmias, and even sudden death.^1^ Although genetic lineage has been identified for some of the cardiomyopathies, familial forms show diverse modes of inheritance according to the expressed phenotype.^1^ Most of the data available about cardiomyopathies refer to an adult population. In this report, we describe a case of a school-aged patient, with an association of restrictive cardiomyopathy and hypertrabeculation, formerly known as non-dilated left ventricular noncompaction (LVNC).

## Case Report

A previously healthy six-year-old girl, without relevant family or consanguinity history, presented with a 24-month history of hyporexia, increase in abdominal circumference, and recurrent emesis; in the last two months with thoracic pain and progressive decrease in functional class. She was found with hepatomegaly approximately 6 cm below the costal margin. An initial echocardiogram revealed severe dilation of the suprahepatic veins and inferior cava vein, with severe biatrial dilation, diastolic dysfunction with a restrictive pattern, and indirect signs of pulmonary hypertension. RCM was suspected and management with diuretics, carvedilol, and enalapril was begun, with improvement in functional class.

Studies were requested to clarify the etiology of RCM, cardiac magnetic resonance reported left ventricular hypertrabeculation with a non-compaction region/compaction region ratio of 4:1, systolic dysfunction, significant hypertrabeculation of the ventricular cavity, dilation of the right ventricle with deterioration of systolic function (Figure 1). In addition, a 24-hour Holter ECG described right and left atrial changes and repolarization disorder in precordial leads. Due to the foregoing, the patient was discussed in a cardiology meeting, considering the coexistence of restrictive cardiomyopathy and hypertrabeculation.

**Figure 1:** Cardiac magnetic resonance: cine SSFP TrusFisp sequence in long axis 2-chamber and short axis where significant hypertrabeculation of the left ventricular cavity is observed, compromising the lateral and inferior and mid-apical walls with a non-compaction region/compaction region ratio of 4.

Anti-failure medical management was started and there was improvement in the New York Heart Association (NYHA) functional classification from III to II. However, after a few months, the patient evidenced clinical deterioration, requiring hospitalization in the intensive care unit for refractory heart failure, and a cardiac tamponade diagnosis was made requiring a pericardial window, support by extracorporeal mechanical oxygenation and subsequent massive cerebral hemorrhage leading to her death.

Written informed consent was obtained from the minor's legal guardian/next of kin, to publish any potentially identifiable images or data in this article. The ethics committee approved the conduct of the study.

### Genomics-based evaluation

Because of the diagnosis of early-onset RCM, the medical genetics team was consulted. Physical examination was unremarkable, and there was no family history of cardiomyopathies or sudden cardiac death. After a careful review of the case, whole exome sequencing (WES) was performed.

A novel heterozygous missense variant in the *FLNC* gene (NM_001458.5) was identified: c.7559C>A, p.Thr2520Asn and confirmed by Sanger sequencing. This substitution converts the threonine codon at position 2520 into asparagine, located in the ROD2 domain in which there is clustering of variants associated mainly with hypertrophic cardiomyopathy. This variant has not been reported in population databases or current medical literature and is classified as likely pathogenic.

Other gene variants identified in this case were: a heterozygous frameshift variant in the *AGK* gene (NM_018238.4): c.675delG, p.Trp225CysfsTer6, classified as pathogenic according to ACMG guidelines; and a heterozygous missense variant in the *PKP2* gene (NM_004572.4): c.1163G>A, p.Arg388Gln, classified as a variant of uncertain significance (VUS).

No other gene variants were identified in this case. The patient's mother (34 years old), father (38 years old), and paternal grandparents (55 and 62 years old) consented to genetic testing, finding that the father was a carrier for both the FLNC and AGK variants (Figure 2). He has a normal echocardiogram and is currently being evaluated by the cardiology team.

**Figure 2:** Pedigree information of the patient and relatives. Black arrow: the proband Red: Restrictive cardiomyopathy (*): Documented evaluation. Standardized human pedigree nomenclature was followed (J Genet Counsel (2008) 17:424–433).

## Discussion

Concerning RCM, the most commonly implicated genes are those encoding sarcomeric proteins, including those for troponin I (*TNNI3*), β-myosin heavy chain (*MYH7*), cardiac α-actin (*ACTC1*), titin (*TTN*), and myosin light-chain genes.^2^ Sporadic and familial cases exist, and up to 30% of patients have a relevant family history with endomyocardial fibrosis being the most frequent cause.^3^ Similarly, hypertrabeculation corresponds to a phenotypic trait rather than a cardiomyopathy by itself.^1^ It is characterized by the presence of prominent left-ventricle trabeculae and deep intertrabecular recesses and has also been linked to sarcomeric genes, most commonly the MYH7 gene.^1^ Therefore, it is debated whether it corresponds to a different cardiomyopathy or a shared morphological trait. It is worth noting, that patients with pathogenic variants in sarcomere genes tend to present an early age onset and a higher incidence of adverse effects.^4^ Other genes linked to hypertrabeculation are ZASP, dystrobrevin, and tafazzin, as well as a single mutation point in the beta-myosin heavy chain gene.^5^

In this case, WES identified 3 different variants: FLNC, AGK, and PKP2 gene variants. Among these, the first variant was of particular interest. *FLNC* gene encodes for actin-binding protein filamin C, one of the three family members found in humans.^6^ Filamin C is composed of 6 domains: an actin-binding domain, ROD1 and ROD2 subdomains, and a dimerization domain at its C-terminus; and it serves as an anchor of sarcolemmal proteins to the cytoskeleton and linkage system cell-cell or cell-extracellular matrix attachments.^6,7^

Mutations in the *FLNC* gene were first associated with the structure and function of skeletal muscle tissue.^6^ A higher prevalence of mutations was found in patients with DCM, followed by HCM.^7^ Few cases have been reported to RCM, with variants located mostly in the ROD1 and ROD2 domains; the variant found in this case is located in ROD2.^7–9^ Other reports have shown non-repeating variants described in the *FLNC* gene among pediatric and adult populations, providing information to suggest a familial linkage.^10,11^ In our case, a novel missense mutation was identified, while a familiar segregation study revealed the father as a carrier of the same variant, currently being a 38-year-old asymptomatic adult. Further studies are needed to incur pathogenic meaning to this mutation and its relation to familial heritage.

Concerning the variants of AGK and PKP2, the first is usually found in Sengers syndrome and an autosomal recessive type of cataract; since the patient has this mutation in a heterozygous state, she is considered a carrier.^12^ The latter, PKP2, a pathogenic heterozygous variant that has been linked to arrhythmogenic cardiomyopathy; in this case, the variant is classified as VUS and does not correlate to the phenotype found, but research is ongoing in the field of cardiovascular genetics to understand the possible contributions of low to medium penetrance gene mutations in cardiomyopathies.^13–15^

As evidenced in our patient, despite the start of medical management, she had a rapid deterioration of her clinical condition due to refractory heart failure with a fatal outcome. Following the guidelines of the European Society of Cardiology, genetic studies could be performed to confirm diagnosis, assess prognosis, select treatment, as part of reproductive counseling, or when the study aims to provide necessary information for a close relative, in our opinion, it should be conducted in every patient whose therapeutic response is not yielding results, in the presence of rapid onset deterioration or multiple types of cardiomyopathies coexisting. Therefore, we consider it relevant to disclose this type of association since a potentially pathogenic variant in FLNC never described before was described, which could explain her evolution and contribute to further studies regarding the topic.
